# To use a simple hernia needle for single-port laparoscopic percutaneous inguinal hernia repair in children: a 5-year experience study

**DOI:** 10.3389/fped.2023.1298643

**Published:** 2024-01-08

**Authors:** Li Zhang, Rui Zhang, Jianfa Zhang, Hailong Hu, Zihan Chen, Yanxiang Fu, Saihua Li

**Affiliations:** ^1^ Department of General Surgery, Yellow River Sanmenxia Hospital, Sanmenxia, China; ^2^ Institute of Medical Technology, Peking University Health Science Center, Beijing, China

**Keywords:** single-port laparoscopic surgery, inguinal hernia, child, pediatric surgery, minimal invasive

## Abstract

**Purpose:**

The aim of this study is to investigate the technique and practical significance of using a simple hernia needle in single-port laparoscopic herniorrhaphy in pediatric patients.

**Methods:**

The study conducted a retrospective analysis of all pediatric patients who underwent treatment for inguinal hernia using single-port laparoscopic herniorrhaphy with a simple hernia needle at Yellow River Sanmenxia Hospital from June 2018 to May 2023. The medical records of all the children were collected, and clinical characteristics, procedural information, and follow-up data were carefully reviewed.

**Results:**

A total of 848 patients underwent inguinal hernia repair, with ages ranging from 7 months to 13 years (2.99 ± 2.49 years), including 756 males and 92 females. A total of 528 cases of unilateral hernia and 310 cases of bilateral hernia were reported, with intra-operative findings revealing contralateral occult hernia in 253 cases. Single-port laparoscopic herniorrhaphy was successfully completed in all patients, with no instances of conversion to open surgery. The mean operation time for unilateral hernia repair was (7.50 ± 4.80) min, while for bilateral hernia repair it was (11.55 ± 7.27) min. Five patients presented with subcutaneous emphysema, while two patients experienced a recurrence of inguinal hernia. No complications, such as scrotal hematoma, trocar umbilical hernia and testicular atrophy, were observed. The duration of the follow-up period ranged from 3 to 24 months.

**Conclusion:**

The promotion and utilization of single-port laparoscopy combined with a simple hernia needle in clinical practice are justified. Our initial investigation indicates that this surgical approach is both safe and dependable for the management of pediatric inguinal hernia. The procedure presents numerous benefits, including the utilization of uncomplicated instruments, straightforward operation, a clear curative impact, minimal tissue damage, rapid recovery, and the absence of scarring.

## Introduction

In children, inguinal hernia is a prevalent and frequently occurring condition, with a higher incidence in boys compared to girls (1). Additionally, the incidence of inguinal hernia in premature infants is greater than in full-term infants (3%–5% vs. 15%) (2). The majority of these conditions are attributed to congenital factors. Some children may experience anorexia, nausea, vomiting, abdominal pain, and other symptoms as a result of an incarcerated hernia mass, which can have implications for the health and nutritional status of children. As the disease progresses, it can also lead to intestinal necrosis (3). Traditionally, pediatric and infant inguinal herniorrhaphy has been performed using an open technique through the inguinal skin crease. While there are multiple iterations of this approach, the fundamental concept involves isolating, ligating, and ultimately excising the hernia sac as near as feasible to the inner inguinal ring. This procedure remains the most frequently conducted elective surgery in the field of pediatric general surgery (4).

The increasing popularity of laparoscopic repair for pediatric inguinal hernia can be attributed to the emergence of minimal access techniques (5, 6). Recent meta-analyses have shown that laparoscopic hernia repair has fewer or comparable testicular complications and does not increase ipsilateral hernia recurrence compared with open hernia repair. Laparoscopic extraperitoneal herniorrhaphy has a lower incidence of metachronous contralateral inguinal hernia than open herniorrhaphy (7).

Nevertheless, laparoscopy presents a lengthier learning curve for novice practitioners. To mitigate the complexity of the procedure, a novel uncomplicated hernia needle is introduced for enhanced ease of use in single-port laparoscopic hernia repair. The utilization of a novel uncomplicated hernia puncture needle device simplifies and expedites laparoscopic hernia repair, and reduces the learning curve associated with this sophisticated technique. Additionally, an assessment of the safety and efficacy was conducted.

## Methods

### Clinical data

This study was retrospective in nature, and therefore informed consent may be waived. This retrospective study comprised 848 pediatric patients who underwent hernia repair utilizing the single-port laparoscopic technique from June 2018 to May 2023. All instances were diagnosed through physical examination and preoperative ultrasound scanning. The study received approval from our institutional review board.

The inclusion criteria are as follows: 1. The patients included in the study had an age range from 6 months to 14 years. 2. The patients were monitored for a period exceeding 3 months following the surgical procedure.

Exclusion criteria: 1. Patients are either younger than 6 months or older than 14 years of age. 2. Patients who were lost to follow-up or observed for less than 3 months were excluded from the analysis. 3. Patients with incarcerated hernias or other conditions necessitating simultaneous surgical intervention.

The collected data encompassed medical history, demographic details, clinical characteristics, surgical treatment outcomes, postoperative complications, and recurrence. All the operations were performed by a senior surgeon.

### Surgical instrument

**Figure 1:**
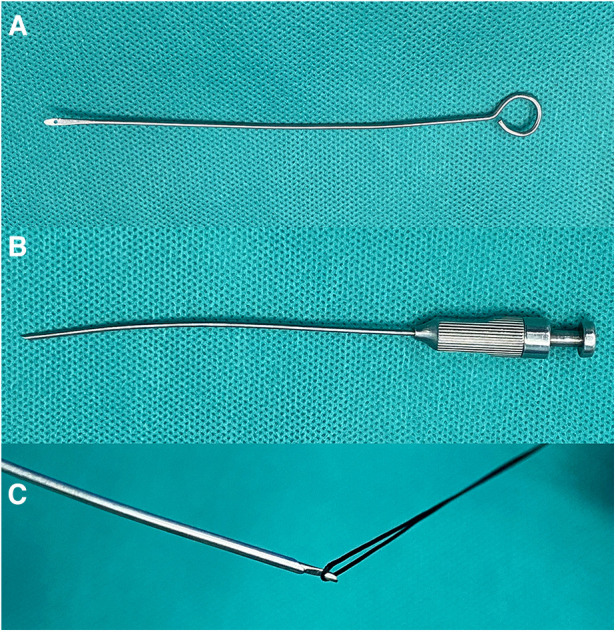
(**A**) hernia needle; (**B**) spring clip needle; (**C**) depress the end of the needle to elongate the crochet, engage the ligating thread.

The equipment used in the pediatric laparoscopy included a 5 mm laparoscope, an arc-shaped hernia needle with a polished sharp end and a drilled hole for silk thread passage (external diameter 1.3 mm, effective working length 15 cm) (Figure 1A), a 1 ml syringe for puncture positioning, and a spring clip needle with a spring-equipped top and a hook groove at the distal end of the needle core. Depress the end of the needle to elongate the crochet, engage the ligating thread, and subsequently release the handle to retract it, allowing for a secure grasp of the thread (Figure 1B,C), 1-0 surgical silk thread.

### Surgical method

**Figure 2:**
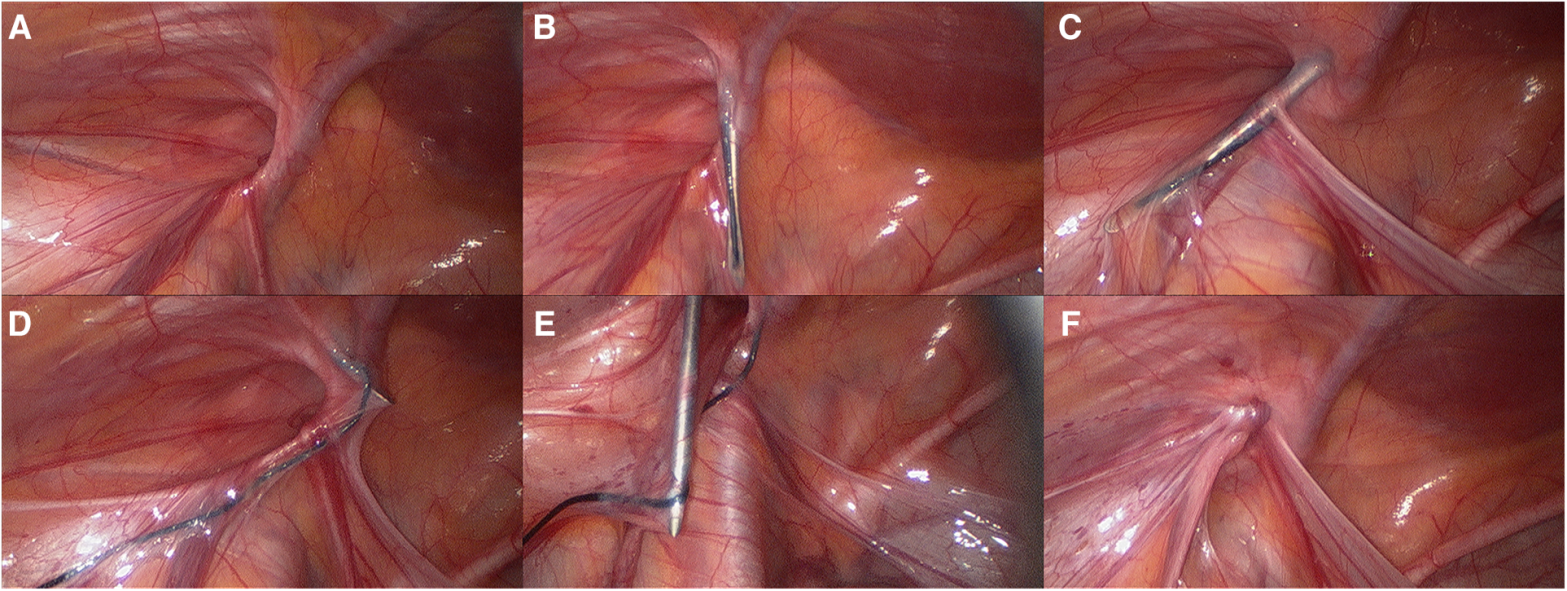
(**A**) identified the position of the hernia; (**B**) hernia needle passed behind the retroperitoneum along the medial edge of the inner ring mouth; (**C**) hernia needle through the vas deferens (or ligamentum teres); (**D**) retracted the wire feeding needle; (**E**) use the hook to catch the end of the line; (**F**) tightening the suture knot.

The patients received intravenous general anesthesia while positioned with their head down or with a soft pad placed under their buttocks. A 5 mm incision was performed at the inferior border of the umbilicus, and CO_2_ pneumoperitoneum was created through puncture with a pneumoperitoneum needle. The pressure was sustained at approximately 8 mmHg (1 mm Hg = 0.133 kPa), and a 5 mm Trocar was inserted. Following abdominal exploration, the researchers identified the position of the hernia ring and examined the shape and dimensions of the hernia ring, as well as the extent of relaxation of the hernia sac, and assess the presence of any concomitant contralateral occult hernia (Figure 2A). A 1 ml syringe needle was utilized to create a puncture mark on the local skin at the upper edge of the inner ring mouth on the anterior abdominal wall. Hernia needle (1-0 silk thread threaded through the needle hole, with both ends left outside the abdominal cavity) was inserted through the puncture point to the retroperitoneum at the upper edge of the inner ring mouth, and subsequently passed behind the retroperitoneum along the medial edge of the inner ring mouth (Figure 2B). Following passage through the vas deferens (or ligamentum teres), the needle tip changed direction towards the lateral aspect, traversed the spermatic cord (or ligamentum teres) vessels towards the midpoint below the inner ring (which may be slightly lateral to access the lateral abdominal wall), and then pierced the peritoneum, extending into the abdominal cavity (Figure 2C). At this juncture, the assistant proceeded to pull one end of the wire, causing the other end to be drawn out through the needle hole of the wire feeder and left within the abdominal cavity. The surgeon carefully retracted the wire feeding needle along the puncture path gap without removing the wire (Figure 2D). Starting from the initial puncture site, insert the spring clip needle into the retroperitoneum of the inner ring, maneuvering behind the retroperitoneum from the lateral edge to the exit point of the abdominal cavity. Once inside the abdominal cavity, push the end of the needle handle to extend the hook, and use the hook to catch the end of the line (Figure 2E). It is challenging to remove the needle and thread after ensuring precision, carefully exiting the needle and the line, and having the assistant expel the air from the scrotum or labia majora before tightening the suture knot. The inner ring was inspected to ensure precise closure (Figure 2F), and the procedure on that side was completed. Simultaneous treatment was administered for hernias on the contralateral side. The incision was sealed using medical adhesive rather than being sutured. Complete surgical video is available in the supplementary document (Supplementary Video S1).

## Result

All patients successfully underwent single-port laparoscopic hernia repair without requiring conversion to open surgery. There were 756 male participants and 92 female participants, with a mean age of (2.99 ± 2.49) years. The average operating time for unilateral hernia patients was (7.50 ± 4.80) minutes, while for bilateral hernia patients it was (11.55 ± 7.27) minutes. Out of 310 patients with bilateral hernia, 253 cases were identified during the operation. During the operation, there was no apparent bleeding, and no damage was observed to the inferior epigastric vessels, spermatic cord vessels, or vas deferens. Subcutaneous emphysema was observed in 5 postoperative patients, while no instances of scrotal hematoma, iatrogenic cryptorchidism, foreign body reaction to suture knots, puncture hernia, or other complications were reported. The children were able to consume food and rise from bed 6 h after regaining consciousness from anesthesia without displaying overt signs of pain. The incision exhibited satisfactory healing with no discernible scarring. The patients were monitored for a period ranging from 3 to 24 months (mean 14.65 ± 5.34 mounth), during which 2 patients were identified as having recurrent inguinal hernia (both had a history of chronic constipation) (Table 1).

**Table 1:** Patients’ characteristics.

Parameters	Observations
No. of patients	838
Mean age	2.99 ± 2.49
Sex	
Male	756
Female	92
Side of hernia	
Unilateral	528 (63.01%)
Bilateral	310 (36.99%)
Contralateral occult	253 (30.19%)
Operating time (min)	
Unilateral	7.50 ± 4.80
Bilateral	11.55 ± 7.27
Hospital stay	1–3 days (1.73 ± 0.32 days)
Follow-up	3–24 months (14.65 ± 5.34 months)
Complications	
Subcutaneous emphysema	5 (0.60%)
Recurrence	2 (0.24%)

## Discussion

Inguinal hernia is one of the most common congenital disorders in children, occurring in 1%–2% of full-term infants and up to 30% of preterm infants (8, 9). The main pathogenesis is the failure of the vaginal process to close, which allows the contents of the abdominal cavity to enter the groin or scrotum through the open vaginal process, resulting in clinical symptoms (10). Currently, open hernia repair is considered the preferred method for treating pediatric inguinal hernias. However, the procedure carries certain risks because it requires opening the inguinal canal and removing the hernia sac. This process increases the likelihood of damaging the vas deferens, spermatic cord vessels, and other associated structures. As a result, complications such as hematoma, iatrogenic cryptorchidism, testicular atrophy, and even infertility may arise (11, 12). Perlstein reported that 2.3%–15% of patients experience testicular dysplasia, atrophy, or even iatrogenic cryptorchidism (13). Furthermore, the vas deferens is vulnerable to mechanical damage. According to Harrison et al., the incidence of vas deferens obstruction on one side was found to be 26.7% in children who had previously undergone open inguinal hernia surgery (14). In contrast, laparoscopic surgery has gained popularity because of its aesthetic benefits, reduced postoperative pain, and ability to minimize damage to the vas deferens and spermatic cord vessels (15). Furthermore, laparoscopy can effectively identify contralateral occult hernias without requiring additional incisions, thereby reducing the need for further surgery to some extent (16, 17). In this study, 253 cases of contralateral occult hernia were found during laparoscopic surgery, representing 47.92% of unilateral hernias diagnosed before surgery. Some patients may develop an inguinal hernia and subsequently need further treatment.

Laparoscopic techniques have been described in numerous versions, ranging from a three-trocar intraperitoneal approach to one or two trocar percutaneous approaches. The technique is constantly advancing with a trend towards increasing the application of extraperitoneal repair and decreasing the use of assistant instruments and trocars.

Single-port laparoscopic high ligation of a hernia sac requires special operating instruments, such as a multi-channel puncture device, Kirschner wire, epidural puncture needle, and abdominal wall suture straight needle. It also requires a scalpel to make a 2–3 mm skin incision at the projection of the inner ring, which may result in skin scars after the operation. During the procedure, the curved wire delivery needle and spring clip needle were used to puncture the abdominal wall directly into the abdominal cavity. The silk thread was then brought out around the inner ring through the needle hole using the wire delivery and spring clip technique, and the incision was sealed with medical glue. The scar at the puncture site could completely disappear within 2 weeks after the surgery. The pointed end of the wire delivery needle is polished, which not only widens the gap of the extraperitoneal channel but also prevents penetration of the peritoneum and damage to the surrounding tissues. It can also smoothly guide the ligature wire into the needle hole, ensuring a safe and quick process. Despite the numerous advantages of this approach, it should be used cautiously in patients with certain types of hernias, as the presence of the organ increases the risk of injury and the complexity of surgery. We have also updated some references (18, 19).

It is important to consider the following points during the operation: (1) when establishing pneumoperitoneum, the umbilical Trocar should be used to blindly enter the abdominal cavity, and the operator and assistant should lift the abdominal wall to prevent accidental injury to the abdominal organs. (2) During the positioning process, it is recommended to advance the laparoscopic lens in order to identify the deep inferior epigastric vessels. Additionally, the use of a 1 ml syringe for the surface positioning of the inner ring is advised to facilitate the success of the puncture needle at one time and to prevent accidental injury. (3) The puncture site may be chosen approximately 1–2 cm above the inner ring, and the needle and thread can be passed through the lower edge of the arcuate, thus avoiding significant blood vessels and nerves in the inguinal region, which enhances safety. (4) It is preferable that the needle does not intersect the vas deferens and spermatic cord vessels in a vertical manner. The instrument can be swung in front of it to widen the gap, and then the peritoneum over the above structures can be gently manipulated. In this procedure, we employ the technique of refining the pointed section of the wire feeding needle to prevent secondary harm, ensuring convenient and safe operation. (5) In the arc-shaped wire feeding needle process, a 1-0 silk thread is passed through the needle hole, with both ends extending outside the abdominal cavity. When the needle pierces the peritoneum and enters the abdominal cavity, one end of the wire is gently pulled by the assistant, while the other end is drawn out through the needle hole and left in the abdominal cavity. (6) Upon grasping the end of the spring clip needle, it is advisable not to withdraw the needle immediately. Hold the outer abdominal segment with one hand and use the other hand to guide the needle into the abdominal cavity. When one perceives the ability to endure a specific level of tension, it is possible to retract the needle in order to prevent the silk thread from slipping. (7) When performing hernia ring ligation in male children, it is important to ensure proper traction of the testis and to apply pressure to the inguinal area to expel air from the scrotum, thereby preventing postoperative scrotal and subcutaneous emphysema. Additionally, care should be taken to avoid ligating the spermatic cord vessels and vas deferens in order to prevent iatrogenic cryptorchidism. (8) After tying and cutting the suture, it is recommended to pull the skin at the puncture point and ensure that the suture knot is left in the preperitoneal space to minimize the sensation of a subcutaneous foreign body. (9) The pneumoperitoneum should be released, with the Trocar being exited first, followed by the lens, in order to prevent the omentum tissue from becoming trapped in the puncture hole with the gas.

## Conclusion

This study presents our findings on the use of single-port laparoscopic hernia repair with a basic hernia needle in 838 pediatric patients with inguinal hernia at our hospital over a 5-year timeframe. The procedure offers the benefits of minimal trauma, rapid recovery, short operation duration, aesthetically pleasing incisions, and a clear therapeutic impact. It is also capable of diagnosing and treating contralateral occult hernia simultaneously, thereby preventing the need for a second operation and the associated pain. Consequently, this approach has the potential for extensive application in the management of pediatric inguinal hernia.

## Data Availability

The original contributions presented in the study are included in the article/**Supplementary Material**, further inquiries can be directed to the corresponding author.
